# Pediatric Online Evidence-Based Medicine Assignment Is a Novel Effective Enjoyable Undergraduate Medical Teaching Tool: A SQUIRE Compliant Study: Erratum

**DOI:** 10.1097/01.md.0000471205.52474.95

**Published:** 2015-08-14

In the article “Pediatric Online Evidence-Based Medicine Assignment Is a Novel Effective Enjoyable Undergraduate Medical Teaching Tool: A SQUIRE Compliant Study,^1^ which appeared in Volume 94, Issue 29 of *Medicine*, Dr. Kotb's and Dr. Lotfi's MD degrees were originally omitted. The article has been corrected online.

## References

[1] Kotb MA Elmahdy HN Khalifa NEDM Pediatric online evidence-based medicine assignment is a novel effective enjoyable undergraduate medical teaching tool: A SQUIRE compliant study. *Medicine* 94 29:e1178. 26200621 10.1097/MD.0000000000001178 PMC4603007

